# Dr. B. T. G.

**Published:** 1874-02

**Authors:** 


*Dr. B. T. G.*—The Senior Editor will soon, if possible, give his views on charity, on the difference between charity and policy, which is often practiced by some under the garbe of charity—a principal. Policy is an artifice, and not unfreqently disgraceful.

